# Antihypernociceptive and Neuroprotective Effects of the Aqueous and Methanol Stem-Bark Extracts of *Nauclea pobeguinii* (Rubiaceae) on STZ-Induced Diabetic Neuropathic Pain

**DOI:** 10.1155/2021/6637584

**Published:** 2021-02-02

**Authors:** Eric Gonzal Tsafack, Marius Mbiantcha, Gilbert Ateufack, Stephanie Flore Djuichou Nguemnang, William Nana Yousseu, Albert Donatien Atsamo, Vanessa Matah Marthe Mba, Carine Flore Adjouzem, Egbe Ben Besong

**Affiliations:** ^1^Laboratory of Animal Physiology and Phytopharmacology, Department of Animal Biology, Faculty of Science, University of Dschang, P.O. Box 67, Dschang, Cameroon; ^2^Laboratory of Animal Physiology, Faculty of Science, University of Yaounde I, P.O. Box 812, Yaounde, Cameroon; ^3^Department of Zoology and Animal Physiology, Faculty of Science, University of Buea, P.O. Box 63, Buea, Cameroon

## Abstract

The greatest common and devastating complication of diabetes is painful neuropathy that can cause hyperalgesia and allodynia. It can disturb psychosocial functioning by increasing levels of anxiety and depression. This work was designed to evaluate the antihyperalgesic, antidepressant, and anxiolytic-like effects of the aqueous and methanol extracts of *Nauclea pobeguinii* stem-bark in diabetic neuropathy induced by streptozotocin in mice. Diabetic neuropathy was induced in mice by the intraperitoneal administration of 200 mg/kg streptozotocin (STZ) to provoke hyperglycemia. *Nauclea pobeguinii* aqueous and methanol extracts at the doses of 150 and 300 mg/kg were administered by oral route, and their effects were evaluated on antihyperalgesic activity (Von Frey filaments, hot plate, acetone, and formalin tests), blood glucose levels, body weight, serum, sciatic nerve proinflammatory cytokines (TNF-*α*, IL-1*β*, and IL-6) and sciatic nerve growth factor (IGF and NGF) rates, depression (open field test, forced swimming test, tail suspension test), and anxiety (elevated plus maze, light-dark box test, social interaction). Oral administration of *Nauclea pobeguinii* stem-bark aqueous and methanol extracts (150 and 300 mg/kg) produced antihyperalgesic, antidepressant, and anxiolytic-like effects in STZ-induced diabetic neuropathic mice. Extracts also triggered a decrease in glycaemia and increased body weight in treated animals. They also significantly (*p* <0.001) reduced tumour necrosis factor alpha (TNF-*α*), interleukin-1 beta (IL-1*β*), and IL-6 and significantly (*p* <0.001) increased nerve growth factor (NGF) and insulin-like growth factor (IGF) in sciatic nerves. The results of this study confirmed that *Nauclea pobeguinii* aqueous and methanol extracts possess antihyperalgesic, antidepressant, and anxiolytic activities and could be beneficial therapeutic agents.

## 1. Introduction

Diabetes that affects about 415 million of adults in the world is a main public health problem that is related to development of pain complications in many tissues of the body, and approximately 16 to 26% of diabetic patients have chronic pain [1]. The greatest common complication among diabetic patients is diabetic neuropathy (DN), but its pathophysiological mechanism is difficult and yet unknown [2]. Diabetic neuropathy is characterized by progressive peripheral neuropathy and causes chronic neuropathic pain and sensory or motor damage in the limits [3]. The mechanism by which hyperglycemia causes painful diabetic neuropathy is still unknown, but elevated blood glucose is at the origin of an increase in advanced glycation end products, protein kinase C isoform activation, mitochondrial dysfunction, and activation of nuclear factor-*κ*B (NF-*κ*B) [4, 5] with important release of proinflammatory cytokines (TNF-*α*, IL-1*β*, and IL-6) [6]. Moreover, DN is characterized by neuronal deterioration and alterations of nerve growth factor (NGF) and insulin-like growth factor (IGF) activity [7]. All these pathways are at the origin of spontaneous pain, irregular sensations such as paresthesia, allodynia, and hyperalgesia [1]. Chronic pain due to diabetic neuropathy is carefully associated with anxiety and depression [8], and the mechanism involves central sensitization and structural variations in the brain [9].

It has been estimated that over 50% of patients who suffer from chronic pain also express clinically diagnosable symptoms of depression and anxiety [8]. Recent treatment of diabetic neuropathy (DN) includes the use of tricyclic antidepressants, selective serotonin reuptake inhibitors, anticonvulsants, opioids and antioxidant protein kinase C inhibitors, COX-2 inhibitors [10, 11], nonsteroidal anti-inflammatory drugs as slight analgesics, and calcium channel ligands such as gabapentin and pregabalin [12]. However, these treatments remain limited due to their partial efficiency and numerous adverse effects [13]. Due to the problems associated with the use of these treatments, the search for natural particles and new therapeutic targets constitutes an orientation in the treatment of diabetic neuropathy, which needs to be focused on. Therefore, there is a necessity to find a new alternative intervention targeting main mechanisms causing nerve injury in DN. Some plants have shown antihyperalgesic properties on streptozotocin-induced diabetic neuropathy in rats.

*Nauclea pobeguinii* (*N. pobeguinii*), plant of the Rubiaceae family, contains tannins, flavonoids, terpenoids, steroids, and alkaloids as constituents [14]. It is used usually to treat stomach and articular pain, fever, and inflammation and to reduce hyperglycemia in Cameroon. Previous studies showed antidiabetic actions of *N. pobeguinii* [15]. The stem-bark water decoctions of *N. pobeguinii* have been described as traditional antihelminthic remedies in Congo [16–18] and for lumbago in Cameroon [19]. Previous work established that aqueous and methanolic stem-bark extracts from *Nauclea pobeguinii* showed analgesic, anti-inflammatory, and antiarthritic (monoarthritis) activity [20]. Tsafack et al. [21] showed that the extracts of this plant are very rich in secondary metabolites, including tannins, flavonoids, steroids, saponins, alkaloids, and terpenoids; the same work showed that aqueous and methanolic extracts of *Nauclea pobeguinii* have immunomodulatory effect (reducing the production of intra- and extracellular reactive oxygen species and decreasing T cell proliferation), anti-inflammatory effect (inhibitory effect on cyclooxygenase, lipoxygenase, and protein denaturation), antioxidant effect (antioxidant capacity on DPPH, ABTS, and NO), and curative effect on polyarthritis model induced by complete Freund's adjuvant injection. In addition, some *N. pobeguinii* constituents (cadambine, 3*α*-dihydro cadambine, and 3*α*,5*α*-tetrahydrodesoxycordifoline) displayed neuroprotective effects on glutamate-induced HT22 cell death [22] and reduced the effects of morphine withdrawal in guinea pigs [23]. Diabetic neuropathy pathogenesis is characterized by an associated hyperalgesia, allodynia, and inflammatory response; we suspect that, due to its analgesic and anti-inflammatory properties, *N. pobeguinii* could ameliorate nerve tissue damage. Additionally, the activity of this plant on depression and anxiety is yet unknown. Thus, in the current study, the antihyperalgesia, antiallodynic, antidepressive, and anxiolytic properties of *N. pobeguinii* aqueous and methanol stem-bark extracts were explored in a model of DN induced by streptozotocin (STZ) in mice.

## 2. Material and Methods

### 2.1. Plant Material and Extraction

Fresh trunk bark of *N. pobeguinii* was collected in Mbalmayo, Nyong-et-So'o Division, in the Central Region of Cameroon in August 2016. After collection, it was authenticated by simple comparison with an existing sample [R. Letouzey sample No. 11,367 (28,335/SRFcam)] at the National Herbarium of Yaounde (Cameroon). Then, the bark was dehydrated in the shade (shelter from the sun) and ground to give a fine powder. Five hundred grams (500 g) of this powder wad soaked in 5 L of distilled water, and the mixture was cooked for 30 minutes, then filtered through filter paper, and evaporated in an oven at 40°C to give 22 g of aqueous extract (4.4% yield). The other part (500 g) was soaked in methanol (2.5 L), and then the mixture was macerated for 72 h and filtered through filter paper. The methanol filtration was concentrated by a rotary evaporator at 65°C under reduced pressure. This process produced 42 g of methanol extract (8.4% yield).

### 2.2. Experimental Animals

In this study, both female and male mice (*Mus musculus*, Swiss) were used. These animals were aged between 12 and 16 weeks and weighed about 27 g. They were reared in the Animal House of the Research Unit of Animal Physiology and Phytopharmacology (URPAP) of the Department of Animal Biology of the Faculty of Science of the University of Dschang (Cameroon). The animals were placed in cages at the rate of 5 animals per cage, under natural conditions of temperature (22 ± 1°C) and humidity (50–80%). The animals received a composed feed and drinking water *ad libitum*. With reference to the work of Adjouzem et al. [24], the experimental procedures have been approved by the local ethics committee and are in accordance with the guidelines for the study of pain in awake animals, published by the NIH Publication No. 85-23 “Principles of Animal Protection,” Laboratory Study of Pain, Ministry of Scientific Research and Technology, which adopted the European Union Guidelines on Animal Care and Experimentation (EWC 86/609).

### 2.3. Drugs and Reagents

The following substances were got from Sigma: streptozotocin (STZ), pregabalin (PGB), fluoxetine, and diazepam. All other elements were of analytical grade and were obtained from the local supplier. Pregabalin, fluoxetine, and diazepam were prepared in normal saline (0.9%).

### 2.4. Induction of Experimental Diabetes by Streptozotocin

In this test, the protocol described previously by Mbiantcha et al. [25] was used. Streptozotocin (200 mg/kg, *i.p.*) was injected into mice to induce experimental diabetes, while control animals received an injection of saline solution (0.9%). Diabetes was confirmed by evaluating glycaemia from a drop of blood got from the tail vein of each animal by using the Accu-Chek Performa Glucometer (Roche Diabetes Care, Inc., Indianapolis, USA) three (3) days after streptozotocin injection. Then, for this study, only mice with a blood glucose level ≥300 mg/dL were used. Two weeks after injection of streptozotocin, treatment was started and continued for 4 weeks, during which mechanical allodynia (Von Frey filaments), thermal hyperalgesia (hot plate), and cold allodynia (acetone) were performed each week.

### 2.5. Antihypernociceptive Activity

To assay the antihypernociceptive activity, 42 mice (36 diabetic and 6 nondiabetic) were carefully chosen and separated into 7 groups with six animals each. Group 1 consisted of nondiabetic mice and received no treatment; group 2 with diabetic mice received normal saline (0.9% *per os*) and was considered as negative control; group 3 with diabetic mice received pregabalin (30 mg/kg, *per os*); groups 4 and 5 (diabetic mice) received *per os* the aqueous extract of *N. pobeguinii* at the doses of 150 and 300 mg/kg, respectively; and groups 6 and 7 (diabetic mice) received orally the methanol extract of *N. pobeguinii* at the doses of 150 and 300 mg/kg, respectively.

### 2.6. Evaluation of the Mechanical Allodynia

In this test, a metallic grid helped as support. Mice were acclimated for 25 min in their environment. To test mechanical allodynia, the plantar surface of each mouse was stimulated by Von Frey filaments (0.04 g) applied in ascending order sequentially on the plantar surface. A positive response was made by rapid withdrawal or flinching. Each mouse received 10 applications, and one application was counted for 10%. The number of applications when an animal reacted was expressed in percentage.

### 2.7. Evaluation of the Thermal Hyperalgesia

To test thermal hyperalgesia, each mouse was placed on the hot plate apparatus (Ugo Basile, Varese, Italy) in constant temperature (54 ± 1°C) during 12 sec approximately with a time limit. The reaction time was recorded from the moment the animal licked its paw and made a jump after placing each mouse on the hot plate [26].

### 2.8. Assessment of Cold Allodynia

To assess cold allodynia, the method described by Mbiantcha et al. [27] was used. The response of diabetic mice to cold stimulus was assessed when acetone was applied five times at 5 min intervals to the plantar surface. Cold allodynia was considered when the animal reacted by rapid withdrawal to two out of five applications representing 40%.

### 2.9. Assessment of Chemical Hyperalgesia

At the end of the treatment, on the 29th day, the mice received the last treatment. One hour (1 h) after treatment, each animal was individually located in a PLEXIGLAS box (20 × 30 × 20 cm) with a 45° angle mirror, and 0.2 ml of formalin (2.5%) was injected under the hind leg paw. Then the licking time of the inoculated paw (translating the nociceptive behavior) was determined constantly until the 60th minute [28].

### 2.10. Sample Collections and Assessment of Proinflammatory Cytokines and Growth Factors Levels

Animals were anesthetized with chloroform individually; the method of cardiac puncture was used to take the blood, following which the blood was introduced into tubes without anticoagulants and then centrifuged at 3000 rpm for 15 min, and the serum was then collected to analyze proinflammatory cytokines (IL-1*β*, TNF*α*, and IL-6). After blood collection, the sciatic nerve of each animal was removed, crushed in PBS-mixed salt at the rate of 0.1 g of organ per 1 ml of buffer, and then centrifuged at room temperature (3000 rpm, 15 min, 4°C) with a centrifuge (Eppendorf 5804 R, Hamburg). Then, the supernatant was taken away to analyze proinflammatory cytokines and growth factors (NGF, IGF) [29].

### 2.11. Evaluation of Antidepressant and Anxiolytic Effects

To determine the antidepressive and anxiolytic properties of the different extracts, the diabetic mice were submitted to various tests.

#### 2.11.1. Assessment of Antidepressive Activity

To evaluate antidepressive activity, diabetic mice (36) and nondiabetic mice (6) were selected for this survey and divided into seven groups of six animals each. Group 1 was used as normal control (nondiabetic group); group 2, diabetic control, received the NaCl (0.9%) as treatment; group 3 was considered positive control and received the fluoxetine (5 mg/kg, *i.p.*); groups 4 and 5 received the aqueous extract, respectively, at the doses of 150 and 300 mg/kg; groups 6 and 7 received the methanol extract at the respective doses of 150 and 300 mg/kg. The animals were treated daily during four weeks, and the following tests were evaluated every week: the open field, tail suspension, and forced swimming tests.

#### 2.11.2. The Open Field Test

For this test, the protocol described by Yi et al. [30] with some modifications was used. The animals were placed in a limp quadrilateral (40 × 60 × 50 cm), and the floor was divided into 12 equal squares. Every mouse was placed in a central region of soil, and the movements were recorded by a video device during 6 minutes.

#### 2.11.3. Tail Suspension Test

The protocol described by Mbiantcha et al. [25] was used to achieve this experimentation. Every mouse was suspended by its tail on the side of a shelf with its head at 5 cm above the floor. The length of immobility was counted when the animals were hung passively and were completely immobile and then recorded during 5 min with the help of a video camera.

#### 2.11.4. Forced Swimming Test

Every mouse was placed in a cylindrical container of 10 cm of diameter and 25 cm of height filled with water (25 ± 1°C) up to the 19 cm mark [25] for 6 minutes, and the time of immobility was recorded by a camcorder during the last 4 minutes. The time of immobility was taken into account when the animals let themselves float in water without movement and kept their head above the water.

#### 2.11.5. Evaluation of Anxiolytic Activity

To evaluate anxiolytic properties, the animals were partitioned and treated as in the previous test (antidepressive test) with the only difference that the positive control in that test received the diazepam (1 mg/kg, *i.p.*) as treatment. These animals were used to conduct the elevated plus maze test, the light-obscurity box test, and the social interaction test.

#### 2.11.6. Elevated Plus Maze Test

The device of labyrinth was composed of two open arms measuring 16 × 5 cm and two closed arms measuring 16 × 5 × 12 cm and joined to a central platform (5 × 5 cm). The labyrinth was raised to a height of 25 cm above the floor. Every mouse was placed individually in the center of the labyrinth with its head turned towards an open arm and then observed during 5 min, and the time spent in the open arms and closed arms was recorded [31].

#### 2.11.7. Light-Dark Box Test

The apparatus here was a quadrilateral limp (45 × 27 × 27 cm) divided into 2 compartments (clear and dark) bounded by a space (7.5 × 7.5 cm) that opens up between the 2 compartments. Every mouse was placed in the center of the clear compartment and the time spent in this compartment, 5 minutes, has been observed and recorded [32].

#### 2.11.8. Social Interaction Test

To carry out this test, a completely open box (22 × 15 × 12 cm) was used. Every mouse having received the treatment was isolated for 1 h and then introduced into its limp with another mouse having received no treatment. After introduction in limps, the time used to fight, to bite, and to lick the neck was counted within 5 minutes [32].

### 2.12. Statistical Analysis

Mean ± standard error of the mean (SEM) was used to present the data. Differences between groups were evaluated by one-way analysis of variance followed by the Tukey posttest, and two-way analysis of variance followed by the Bonferroni posttest. At *p* <0.05, the differences were significant and were materialized by *a*, *b*, and *c* against the normal control and *α*, *β*, and *λ* against the negative control.

## 3. Results

### 3.1. Antihyperalgesic Activities

#### 3.1.1. Effects of the Extracts of *N. pobeguinii* on the Mechanical Allodynia

**Figure 1:**
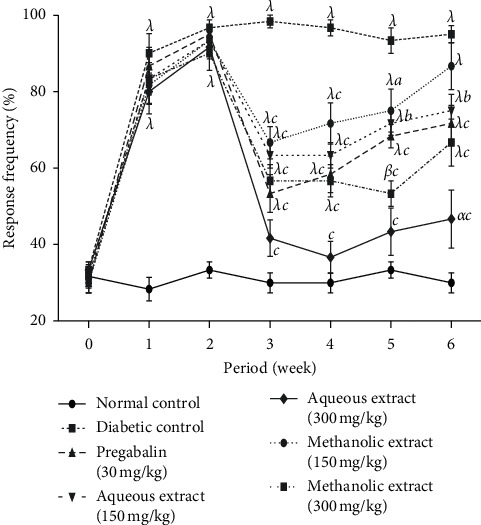
Antihyperalgesic effects of the aqueous and methanol extracts of *N. pobeguinii* on mechanical allodynia induced by Von Frey filaments. Data are expressed as the mean ± SEM of six animals per experimental group, compared by two-way analysis of variance followed by Bonferroni's test; ^*α*^*p* <0.05, ^*β*^*p* <0.01, and ^*λ*^*p* <0.001 mean significantly different from the normal control group (nondiabetic); and ^*a*^*p* <0.05, ^*b*^*p* <0.01, and ^*c*^*p* <0.001 mean significantly different from the diabetic control group.

The effects of the aqueous and methanol extracts of *N. pobeguinii* on the mechanical allodynia are represented in Figure 1. STZ injection produced a significant (*p* <0.001) increase in mechanical response to Von Frey filaments in all groups compared to normal control group, two weeks after STZ injection. However, the administration of the different extracts as well as pregabalin significantly (*p* <0.05; *p* <0.01; *p* <0.001) reduced the mechanical allodynia during the whole period of treatment. A maximal inhibition of 66.4% was observed with the dose of 300 mg/kg of the aqueous extract on the fourth week.

#### 3.1.2. Effects of the Extracts of *N. pobeguinii* on Thermal Hyperalgesia

**Figure 2:**
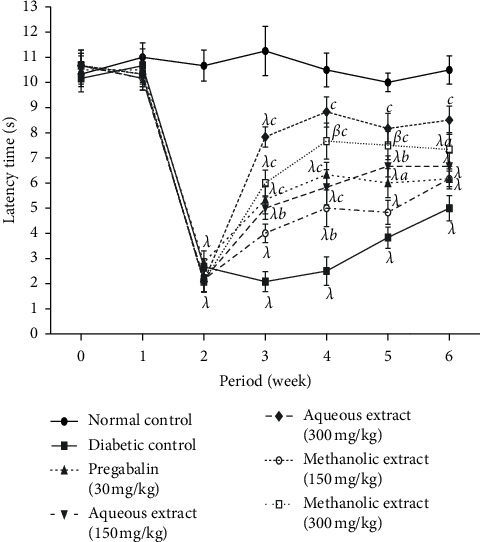
Antihyperalgesic effects of the aqueous and methanol extracts of *N. pobeguinii* on thermal hyperalgesia induced by hot plate (54 ± 1°C). Data are expressed as the mean ± SEM of six animals per experimental group, compared by two-way analysis of variance followed by Bonferroni's test; ^*β*^*p* <0.01 and ^*λ*^*p* <0.001 mean significantly different from the normal control group (nondiabetic); and ^*a*^*p* <0.05, ^*b*^*p* <0.01, and ^*c*^*p* <0.001 mean significantly different from the diabetic control group; s: seconds.

The results got on the thermal hyperalgesia induced by the hot plate of the aqueous and methanol extracts of *N. pobeguinii* are shown in Figure 2. It is observed in this figure that streptozotocin administration significantly (*p* <0.001) decreased the time of latency of the paw among all mice compared to normal animals two weeks after injection of streptozotocin. Aqueous and methanol extracts as well as the pregabalin administration (30 mg/kg) significantly (*p* <0.05; *p* <0.01; *p* <0.001) reduced hot thermal hyperalgesia compared to negative control group animals. This effect began in the first week after treatment and was maintained during the whole period of treatment. However, a maximal reduction was observed in the fourth week with the aqueous extract (300 mg/kg).

#### 3.1.3. Effects of the Extracts of *N. pobeguinii* on Cold Allodynia

**Figure 3:**
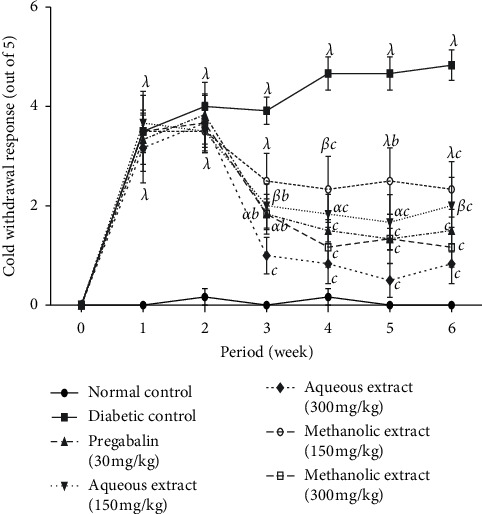
Antihyperalgesic effects of the aqueous and methanol extracts of *N. pobeguinii* on cold allodynia induced by acetone. Data are expressed as the mean ± SEM of six animals per experimental group, compared by two-way analysis of variance followed by Bonferroni's test; ^*α*^*p* <0.05, ^*β*^*p* <0.01, and ^*λ*^*p* <0.001 mean significantly different from the normal control group (nondiabetic); and ^*b*^*p* <0:01 and ^*c*^*p* <0.001 mean significantly different from the diabetic control group.

Figure 3 presents the results obtained for the assessment of the antihyperalgesia effect of aqueous and methanol extracts of *N. pobeguinii* on the response to the cold stimulus caused by acetone. It ensures that animals that received streptozotocin injection developed significantly (*p* <0.001) allodynia characterized by the withdrawal of the paw following the administration of the acetone two weeks after injections of streptozotocin in comparison to normal animal groups. However, extract administration significantly (*p* <0.05; *p* <0.01; *p* <0.001) reduced the cold allodynia from the third to the sixth week compared to negative control animals. A maximal reduction was obtained with the aqueous extract (300 mg/kg) during the fifth week.

#### 3.1.4. Effects of the Extracts of *N. pobeguinii* on Chemical Hyperalgesia

**Figure 4:**
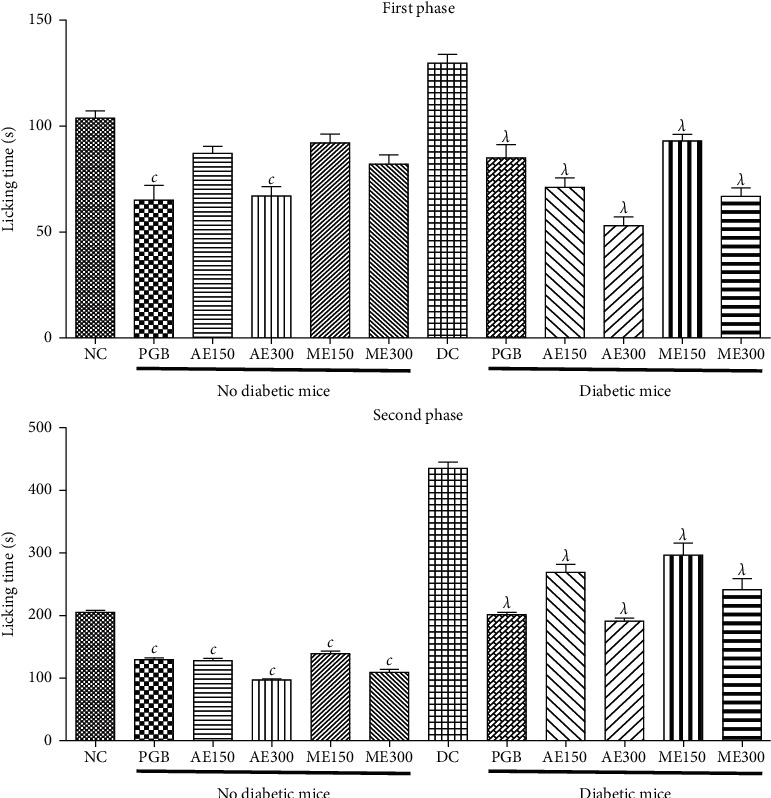
Effects of the aqueous and methanol extracts of *N. pobeguinii* on the two phases of the pain induced by formalin. Data are expressed as the mean ± SEM of six animals per experimental group, compared by one-way analysis of variance followed by Tukey test; ^*λ*^*p* <0.001 means significantly different from the normal control group (nondiabetic); and ^*c*^*p* <0.001 means significantly different from the diabetic control group.

Figure 4 shows a double-phase response observed in diabetic and nondiabetic mice after formalin injections. In the first phase, aqueous extract (300 mg/kg) as well as pregabalin (5 mg/kg) showed significant (*p* <0.001) inhibitory effects on nondiabetic and diabetic mice compared, respectively, to negative control nondiabetic mice and negative control diabetic mice. Thus, a maximal inhibitory effect of aqueous extract was 30.76% in nondiabetic mice. In diabetic mice, the aqueous extract (300 mg/kg) showed a maximal inhibitory effect of 54.82%, while the methanol extract at the same dose showed a maximal inhibitory effect of 44.18%.

However, the aqueous and methanol extracts at all doses tested as well as pregabalin showed significant (*p* <0.001) inhibitory effects on nondiabetic and diabetic mice compared to negative control nondiabetic mice and negative control diabetic mice, respectively, in the second phase (Figure 4). Inhibitory effects of 50.28% and 44.12% were obtained with the aqueous and methanol extracts in nondiabetic mice, respectively. Thus, in diabetic mice, the aqueous extract (300 mg/kg) showed an inhibitory effect of 55.58%, while the methanol extract (300 mg/kg) showed 45.08% in diabetic mice.

#### 3.1.5. Effects of the Extracts of *N. pobeguinii* on the Blood Glucose Level

Table 1 presents the effects of aqueous and methanol extracts of *N. pobeguinii* on the blood glucose levels of the animals after treatment with the streptozotocin. It is observed from this table that, after administration of streptozotocin, the sugar in the blood significantly (*p* <0.001) increased for all animals in comparison to the animals not having received the streptozotocin. However, the administration of the different extracts at all doses as well as pregabalin decreased significantly (*p* <0.001) the blood sugar compared to diabetic control animals. This effect began one week after the beginning of the treatment and was maintained during the whole period of treatment.

#### 3.1.6. Effects of the Extracts of *N. pobeguinii* on the Body Weight

Table 2 shows the effect of the extract of *N. pobeguinii* on body weight variation after injections of the STZ. It can be noticed again that the injection of STZ entailed a significant reduction (*p* <0.05; *p* <0.01; *p* <0.001) of the body weight in all animals from the second week compared to nondiabetic animals. After treatment with aqueous and methanol extracts of *N. pobeguinii*, the body weight of animals increased meaningfully (*p* <0.01; *p* <0.001) compared to diabetic control animals.

#### 3.1.7. Effects of the Extracts of *N. pobeguinii* on the Proinflammatory Cytokine Level

**Figure 5:**
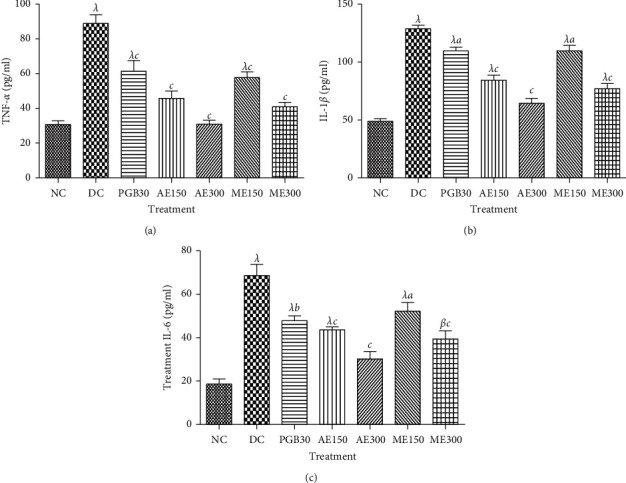
Effects of the aqueous and methanol extracts of *N. pobeguinii* on the levels of TNF-*α* (a), IL-1*β* (b), and IL-6 (c) in the serum of diabetic mice. Data are expressed as the mean ± SEM of six animals per experimental group, compared by one-way analysis of variance followed by Tukey test; ^*β*^*p* <0.01 and ^*λ*^*p* <0.001 mean significantly different from the normal control group (nondiabetic); and ^*a*^*p* <0.05, ^*b*^*p* <0.01, and ^*c*^*p* <0.001 mean significantly different from the diabetic control group. NC: normal control, DC: diabetic control, PGB30: mice that received 30 mg/kg/day pregabalin, AE150: mice that received 150 mg/kg/day of aqueous extract, AE300: mice that received 300 mg/kg/day of aqueous extract, ME150: mice that received 150 mg/kg/day of methanol extract, ME300: mice that received 300 mg/kg/day methanol extract, TNF: tumour necrosis factor, IL: interleukin.

**Figure 6:**
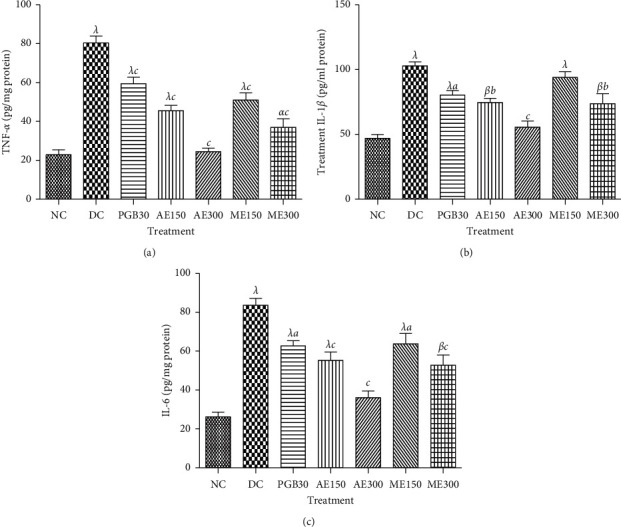
Effects of the aqueous and methanol extracts of *N. pobeguinii* on the levels of TNF-*α* (a), IL-1*β* (b), and IL-6 (c) in the sciatic nerves of diabetic mice. Data are expressed as the mean ± SEM of six animals per experimental group, compared by one-way analysis of variance followed by Tukey test; ^*α*^*p* <0.05, ^*β*^*p* <0.01, and ^*λ*^*p* <0.001 mean significantly different from the normal control group (no diabetic); and ^*a*^*p* <0.05, ^*b*^*p* <0.01, and ^*c*^*p* <0.001 mean significantly different from the diabetic control group. NC: normal control, DC: diabetic control, PGB30: mice that received 30 mg/kg/day pregabalin, AE150: mice that received 150 mg/kg/day of aqueous extract, AE300: mice that received 300 mg/kg/day of aqueous extract, ME150: mice that received 150 mg/kg/day of methanol extract, ME300: mice that received 300 mg/kg/day methanol extract, TNF: tumour necrosis factor, IL: interleukin.

In Figures 5 and 6, it is observed that injection of streptozotocin showed a significant (*p* <0.001) increase in proinflammatory cytokines (IL-1*β*, TNF-*α*, and IL-6) on serum and sciatic nerve in nontreated diabetic mice compared to nondiabetic mice. However, administration of aqueous and methanol extracts of *N. pobeguinii* at all doses as well as pregabalin reduced significantly (*p* <0.05; *p* <0.001) proinflammatory cytokines compared to the diabetic untreated control group. The maximum reductions of 58.88%, 46.15%, and 55.88% were obtained with aqueous extract (300 mg/kg) on TNF-*α*, IL-1*β*, and IL-6, respectively, in serum (Figure 5). Concerning the sciatic nerve, the maximum inhibitions of 70.62% (TNF-*α*), 42.85% (IL1-*β*), and 58.88% (IL-6) were obtained with the dose of 300 mg/kg of the aqueous extract (Figure 6).

#### 3.1.8. Effects of the Extracts of *N. pobeguinii* on the Growth Factors Levels

**Figure 7:**
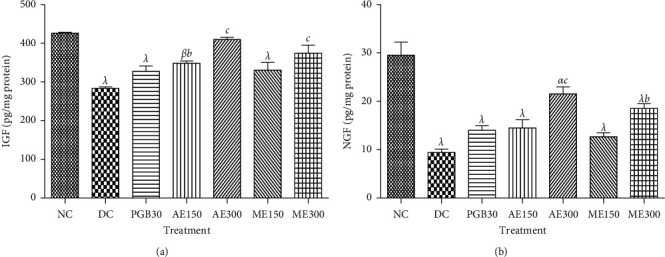
Effects of the aqueous and methanol extracts of *N. pobeguinii* on the levels of IGF (a) and NGF (b) in the sciatic nerves of diabetic mice. Data are expressed as the mean ± SEM of six animals per experimental group, compared by one-way analysis of variance followed by Tukey test; ^*α*^*p* <0.05, ^*β*^*p* <0.01, and ^*λ*^*p* <0.001 mean significantly different from the normal control group (nondiabetic); and ^*b*^*p* <0.01 and ^*c*^*p* <0.001 mean significantly different from the diabetic control group. NC: normal control, DC: diabetic control, PGB30: mice that received 30 mg/kg/day pregabalin, AE150: mice that received 150 mg/kg/day of aqueous extract, AE300: mice that received 300 mg/kg/day of aqueous extract, ME150: mice that received 150 mg/kg/day of methanol extract, ME300: mice that received 300 mg/kg/day methanol extract, IGF: insulin-like growth factor, NGF: nerve growth factor.

Figure 7 shows the effects of the aqueous and methanol extracts of *N. pobeguinii* on growth factors (NGF and IGF) levels in sciatic nerve. It has been observed that the level of growth factors in the sciatic nerve was increased significantly (*p* <0.001) in nontreated diabetic mice compared to nondiabetic mice. Thus, treatment of animal with the aqueous and methanol extract of *N. pobeguinii* significantly (*p* <0.01; *p* <0.001) decreased NGF and IGF levels, compared to nontreated diabetic mice. Maximum decreases of 48.27% and 80.64% were obtained with the aqueous extract (300 mg/kg) on the level of IGF and NGF, respectively.

### 3.2. Diabetic Painful and Neuropharmacological Activity

#### 3.2.1. Antianxiety Activities

**Figure 8:**
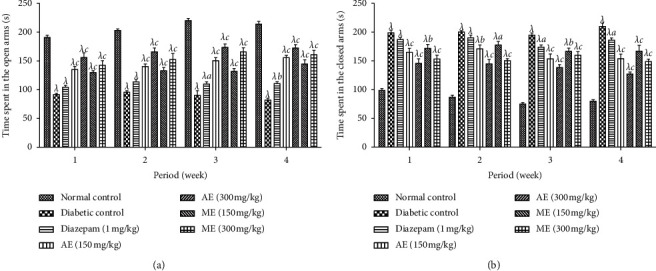
Effects of the aqueous and methanol extracts of *N. pobeguinii* on the absolute time spent in the open arms (a) and closed arms (b) during 6 min of exposure to the elevated plus maze. Data are expressed as the mean ± SEM of six animals per experimental group, compared by two-way analysis of variance followed by Bonferroni's test; ^*λ*^*p* <0.001 means significantly different from the normal control group (nondiabetic); and ^*a*^*p* <0.05, ^*b*^*p* <0:01, and ^*c*^*p* <0.001 mean significantly different from the diabetic control group; s: seconds.

*(1) Elevated plus Maze Test*.  Figure 8 shows the effects of the aqueous and methanol extracts of *N. pobeguinii* on the time spent in the open arm (A) and closed arm (B) during 6 minutes for elevated plus maze tests in diabetic mice. It is observed that the animals that received the two extracts at all doses tested as well as diazepam significantly increased (*p* <0.01; *p* <0.001) the time spent in the open arms and significantly reduced (*p* <0.01; *p* <0.001) the time spent in the closed arms, compared to the untreated diabetic animals. These activities were observed during the whole period of treatment, and the dose of 300 mg/kg of the aqueous extract caused the most important effect.

**Figure 9:**
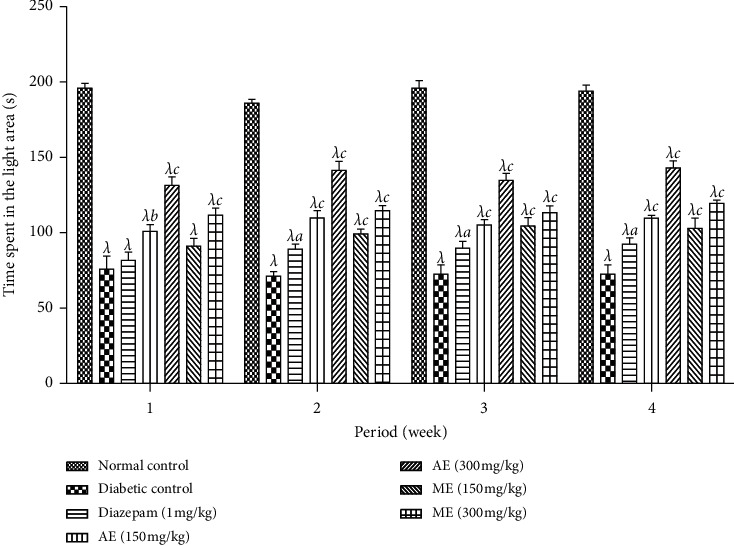
Effects of the aqueous and methanol extracts of the *N. Pobeguinii* on the absolute time spent in the open (white/light) compartment during 5 min of exposure to the light-dark box test. Data are expressed as the mean ± SEM of six animals per experimental group, compared by two-way analysis of variance followed by Bonferroni's test; ^*λ*^*p* <0.001 means significantly different from the normal control group (nondiabetic); and ^*a*^*p* <0.05 and ^*c*^*p* <0.001 mean significantly different from the diabetic control group; s: seconds.

*(2) Light-Dark Box Test*. The time spent in the illuminated area during 6 minutes of exhibition in illuminated and nonilluminated limps of the diabetic animals that received the aqueous and methanol extracts of *N. pobeguinii* is illustrated in Figure 9. It is shown in this figure that the time spent by the diabetic animals in the illuminated area significantly (*p* <0.001) reduced compared to the nondiabetic animals. The treatment of the mice with the aqueous and methanol extracts of *N. pobeguinii* and diazepam (1 mg/kg) provoked a meaningful (*p* <0.05; *p* <0.01; *p* <0.001) increase in the time spent in the illuminated area from the beginning to the end of the treatment in comparison to the nontreated diabetic mice.

**Figure 10:**
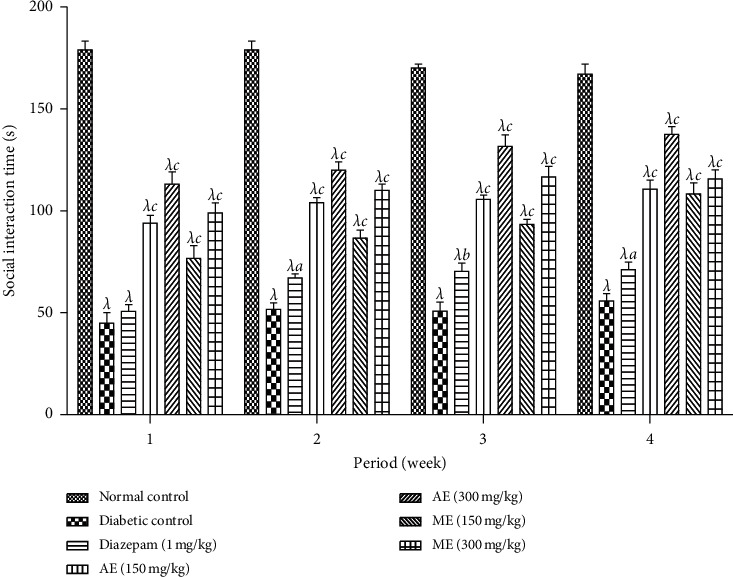
Effects of the aqueous and methanol extracts of *N. pobeguinii* on the absolute time for social interaction during 5 min. Data are expressed as the mean ± SEM of six animals per experimental group, compared by two-way analysis of variance followed by Bonferroni's test; ^*λ*^*p* <0.001 means significantly different from the normal control group (nondiabetic); and ^*a*^*p* <0.05, ^*b*^*p* <0.01, and ^*c*^*p* <0.001 mean significantly different from the diabetic control group; s: seconds.

*(3) Social Interaction Test*.  Figure 10 presents the effect of the aqueous and methanol extracts of *N. pobeguinii* on the length of social interaction of the diabetic mice during 6 minutes. The figure indicates a significant (*p* <0.001) reduction of the social interaction length among diabetic mice in comparison to normal mice. However, the treatment of the mice at all doses tested of the aqueous and methanol extracts as well as diazepam significantly (*p* <0.001) increased this parameter, compared to the nontreated diabetic mice.

#### 3.2.2. Antidepressive Activities

**Figure 11:**
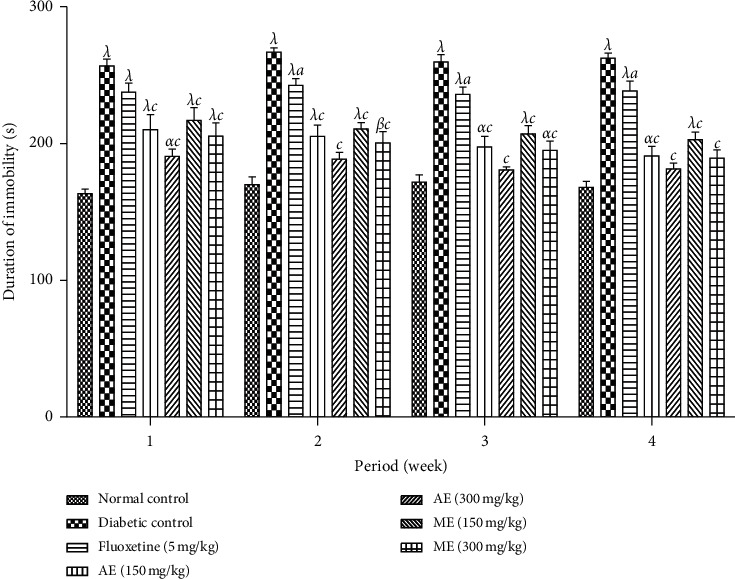
Effects of the aqueous and methanol extracts of *N. pobeguinii* on the immobility time of mice in the FST during 5 min. Data are expressed as the mean ± SEM of six animals per experimental group, compared by two-way analysis of variance followed by Bonferroni's test; ^*α*^*p* <0.05, ^*β*^*p* <0.01 and ^*λ*^*p* <0.001 mean significantly different from the normal control group (nondiabetic); and ^*a*^*p* <0.05 and ^*c*^*p* <0.001 mean significantly different from the diabetic control group; s: seconds.

*(1) Forced Swimming Test*.  Figure 11 shows the effects of the aqueous and methanol extracts of *N. pobeguinii* on the immobility time of the diabetic mice in the swimming forced test during 6 minutes. It is observed in this phase that the mice that received either extract of *N. pobeguinii* at all tested doses as well as the fluoxetine at the dose of 5 mg/kg reduced significantly (*p* <0.05; *p* <0.001) the time of immobility in comparison to the animals of the negative control group.

**Figure 12:**
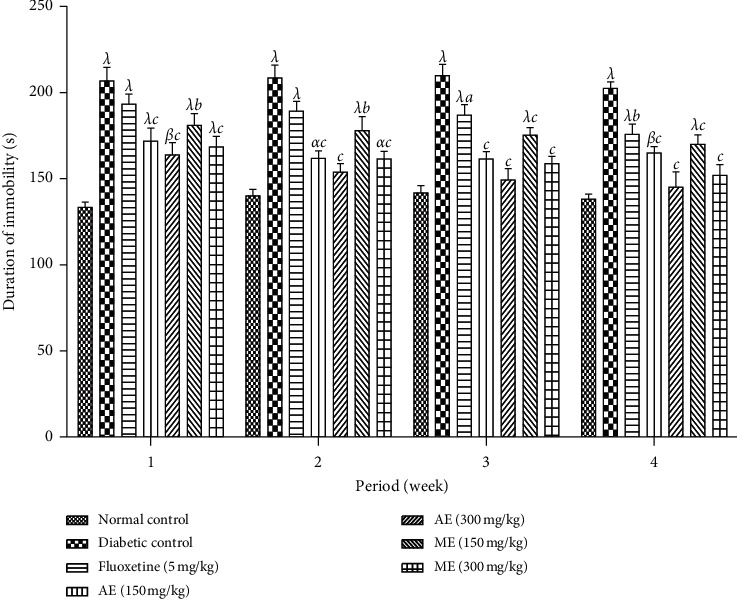
Effects of the aqueous and methanol extracts of *N. pobeguinii* on the immobility time of mice in the TST during 5 min. Data are expressed as the mean ± SEM of six animals per experimental group, compared by two-way analysis of variance followed by Bonferroni's test; ^*α*^*p* <0.05, ^*β*^*p* <0.01, and ^*λ*^*p* <0.001 mean significantly different from the normal control group (nondiabetic); and ^*a*^*p* <0.05 and ^*c*^*p* <0.001 mean significantly different from the diabetic control group; s: seconds.

*(2) Tail Suspension Test*. The immobility time of the mice submitted to the tail suspension test of the diabetic mice treated with either extract of *N. pobeguinii* is presented in Figure 12. In this phase, there is a significant reduction (*p* <0.01; *p* <0.001) of immobility time of the animals that received the different doses of the aqueous and methanol extracts of *N. pobeguinii* during the whole time of treatment in comparison to the nontreated diabetic animal. The mice treated with fluoxetine significantly (*p* <0.05; *p* <0.01) reduced immobility time compared to the negative control animals from the third week of treatment, whereas a nonsignificant reduction was observed during the first two weeks of treatment.

**Figure 13:**
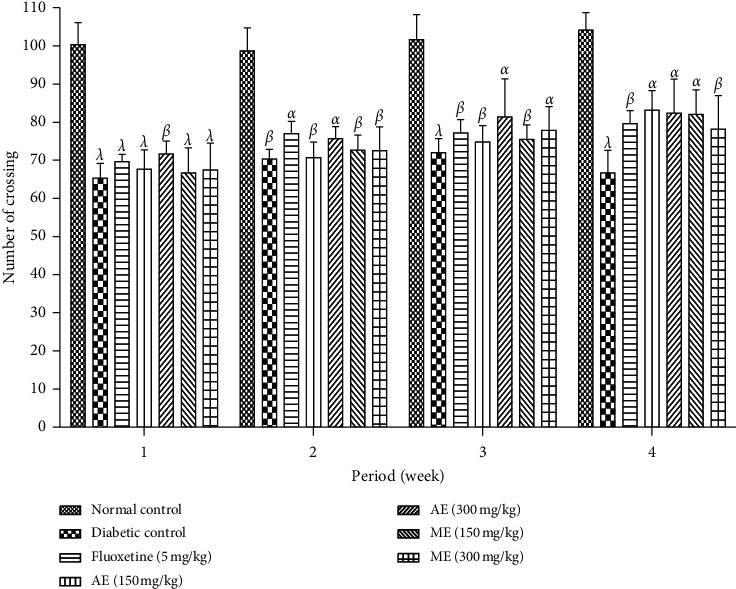
Effects of the aqueous and methanol extracts of *N. pobeguinii* on the locomotor activity of mice in the OFT during 5 min. Data are expressed as the mean ± SEM of six animals per experimental group, compared by two-way analysis of variance followed by Bonferroni's test; ^*α*^*p* <0.05, ^*β*^*p* <0.01, and ^*λ*^*p* <0.001 mean significantly different from the normal control group (nondiabetic).

*(3) Open Field Test*. The effects of the aqueous and methanol extracts of *N. pobeguinii* on the number of passages of the mice on open land are presented in Figure 13. In this phase, it is observed that there was a significant reduction (*p* <0.05; *p* <0.01; *p* <0.001) in the number of passages of mice on open land among all diabetic animals compared to the nondiabetic animals. However, when the animals received different doses of the extract, as well as the reference medicine, a nonsignificant increase in the number of passages of the mice was recorded compared to the nontreated diabetic mice.

## 4. Discussion

The main objective of this study was to evaluate the antihyperalgesia and neuropharmacological properties of the aqueous and methanol extracts of *N. pobeguinii* stem on streptozotocin-induced painful diabetic neuropathy in mice. The previous studies on *N. pobeguinii* showed that this plant possesses some in vitro anti-inflammatory properties on protein denaturation, cyclooxygenase, and 5-lipoxygenase inhibition; ROS production; and cell proliferation and antioxidant properties against the radical DPPH and ABTS [21]. *In vivo*, this plant showed analgesic properties on the two phases of pain caused by formalin [20] and poly arthritis caused by CFA in rats [21].

In fact, the greatest common difficulty related to diabetes mellitus is neuropathy, and the recurrent and devastating consequence of diabetic neuropathy is pain [33]. It has been demonstrated that hyperalgesia (changes in pain perception, improved sensitivity to noxious stimuli), allodynia (irregular pain sensitivity before nonpainful stimuli), and inflammatory mediators (cytokines) characterize a chronic or persistent neuropathic pain perceived in diabetic rats [12]. To evaluate the effectiveness of probable analgesic agents, a painful diabetic model induced by STZ on mice has been progressively used for this motive, as a model of neuropathy [27, 29]. The Von Frey filament test, the hot plate test, the acetone test, and the formalin test that characterize, respectively, mechanical allodynia, thermal hyperalgesia, cold allodynia, and chemical hyperalgesia were selected to evaluate the antihyperalgesic properties of aqueous and methanol extracts of *N. pobeguinii* stem-bark in the present study. The findings evidently show that, by the oral route, prolonged administration of aqueous and methanol extracts created in all models of pain tests in diabetic rats important antihyperalgesic and antiallodynic activities. Since it has the improvement of showing a persistent response period that eases experimental studies and involvement, the formalin test is one of the finest assessments to evaluate allodynia and/or hyperalgesia in diabetic mice [27]. After STZ induced diabetes in mice, once formalin is inoculated into the paw, these animals develop a double-phase nociceptive overstated behavior [34]. In this study, through the two stages of the formalin test, both extracts of *Nauclea pobeguinii* had an antihyperalgesic response. Furthermore, in the nondiabetic rats, a significant activity was also acquired by the aqueous extract (300 mg/kg) in the first phase and both extracts at all tested doses in the early phase of the formalin test. That effect suggests that this plant extract could be disturbing the central and peripheral mechanisms related to the diverse phases of the formalin test [27]. Lastly, the study shows that, at the same doses used in this study, these extracts possess analgesic activities on the two phases of the formalin painful model and anti-inflammatory properties on acute and chronic inflammation induced by formalin in rats [20]. On the other hand, a previous study also shows that *N. pobeguinii* stem-bark extract possesses inhibitory activities on COX-2, LIPOX-5, proteins denaturation, and ROS production [21]. It is possible that the antihyperalgesic and antiallodynic effects of aqueous and methanol extracts of *N. pobeguinii* could be linked to the capacity of these extracts to inhibit these different pathways by inhibiting several nociceptive and proinflammatory mediators (PGE2, cytokines, bradykinin, P substance and glutamate, nitric oxide) [35]. However, in the pathophysiology of diabetic neuropathy such as STZ-induced neuropathic pain in mice, hyperglycemia plays a vital part in the release of these mediators [4]. To confirm if the antihyperalgesic and antiallodynic effects of *N. pobeguinii* extracts used in this study can be linked to their antihyperglycemic properties, the serum glucose level of mice was evaluated.

In the present work, hyperglycemia was maintained during the experimental period of several weeks after STZ injection into healthy mice in all animals. Remarkably, as compared with the untreated diabetic mice, the antihyperalgesic and antiallodynic effects of *N. pobeguinii* extracts in diabetic animals were supplemented by an important decrease in serum glucose levels. Then, the interesting antihyperalgesic effects of these compounds in diabetic mice may be linked to the diminution of hyperglycemia [25, 27]. Hyperglycemia observed among the control negative animals has been accompanied by a significant loss of weight. Indeed, it has been shown that STZ injection on mice is at the origin of beta pancreatic cells destruction and therefore the reduction of the secretion of insulin [36]. This deficiency in insulin involves a decrease of glucose capture by the cells and an increase of the protein's catabolism leading to a loss of weight [37, 38]. *N. pobeguinii* aqueous and methanol extract administration during the last two weeks provoked a decrease in loss of weight, compared to the diabetic control group. This capacity of the extract to slow down the loss of weight would be probably due to its antihyperalgesic and antiallodynic effects.

To many forms of neuropathic pain, inflammatory intermediaries such as cytokines have been linked [39]. In fact, in the process of neuropathic pain, immune cells play a vital role [40]. These cells are stimulated under hyperglycemic conditions in the spinal cord [41, 42]. Several studies have revealed that, in the spinal cord, activated microglia (resident macrophages of the central nervous system) play a crucial role in neuropathic pain through the release of proinflammatory cytokines, which are common mediators of allodynia and hyperalgesia [43]. Additionally, in rats, recent statement shows that painful neuropathy that results from STZ-induced type 1 diabetes is related to the release of proinflammatory cytokines such as IL-1*β*, IL-6, and TNF-*α* in serum and sciatic nerve [25] associated with allodynia and hyperalgesia [38] as a consequence of hyperglycemia and insulin resistance installation [26]. Zuo et al. [44] and Sweitzer et al. [45] showed that, in people suffering from diabetic neuropathy, the levels of NGF, lipoxygenase, cyclooxygenase, and prostaglandins are very high. In addition, in clinical conditions in patients with chronic pain such as rheumatoid arthritis, cancer pain, or neuropathic pain, the growth factor (NGF and IGF) levels are very high, which results in significant activation of nociceptors, thus enabling the maintenance of chronic neuropathic pain [46–48]. In the present study, the capability of aqueous and methanol extracts of *N. pobeguinii* to reduce proinflammatory cytokines (IL-1*β*, IL-6, and TNF-*α*) and growth factors (NGF and IGF) was assessed in serum and sciatic nerve, and the findings showed that these extracts decreased these proinflammatory cytokines and nerve growth factor following STZ injection compared to negative control mice. These results could suggest that these extracts can interfere with the immune system cells, particularly microglia cells. These results are similar to those of our recent work which showed that the extracts of *N. pobeguinii* possessed immunomodulatory activities on intracellular and extracellular ROS production, T cells proliferation capacity, antioxidant capacity, and anti-inflammatory capacity through the inhibition of protein denaturation, COX, and 5-LOX activity [21]; these extracts are rich in flavonoids, saponins, and terpenoids. Among the compounds already isolated, we have *p*-coumaric acid and resveratrol [15, 49]. The presence of these compounds may justify the activities of this plant, since *p*-coumaric acid and resveratrol reduced TNF*α*, IL-1*β*, and IL-6 levels in collagen-induced arthritis [50–52] and significantly inhibited iNOS, COX-2, IL-1*β*, and TNF-*α* expression through blocking NF-kB and MAPKs signaling pathways [53, 54]. Moreover, nuclear factor *κ*B (NF-*κ*B), which is a protein transcription factor of a number of diverse inflammatory and immune intermediaries, has various useful paths, precisely, inflammation, immunity, pain, proliferation, and hyperglycemia [29]. Stimulation of this transcription factor by release of the complex I*κ*B/NF-*κ*B activates the transcription of cytokines (TNF-*α*, IL-1*β*, IL-6), and nerve growth factor can be used as an indicator of the state of inflammation [39]. In estimation of the previous findings, it is clear that aqueous and methanol extracts of *N. pobeguinii* activity on cytokines levels obtained in this work is correlated to a modulatory effect on nuclear factor NF-*κ*B activity [25].

Animals develop a state of anxiety and depression added to the augmented sensitivity to pain in the characteristics of diabetic neuropathy [25, 55]. In fact, neuropathic chronic pain is commonly associated with the depressive and anxious syndrome [56] that can increase in animal models [55], and the mechanism implies a central sensitization and structural modification of the brain [55, 57]. Depression can be evaluated by TST, FST, and OFT, and anxiety can be evaluated by EPMT, LDBT, and SIT [58, 59]. The greatest, usually pharmacological, approach used to evaluate antidepressant activity depends on its easiness to use, consistency, crossways, laboratories, and its ability to identify; a broad range of clinically antidepressants are TST and FST [60]. To assess depression and antidepressant compounds, these tests are still the most widely standard [61]. It is supposed that using these two models can provide paired and joining information on the activities of potential antidepressants according to Cryan et al. [60]. A behavioral depression that produces a depressive state explains the immovability displayed by test animals in these models [62]. In the present work, *N. pobeguinii* aqueous and methanol extracts were used to assess the immobility time of STZ-induced neuropathy diabetic mice in TST and FST. The results obtained showed that these extracts decreased the immobility time in all these tests compared to the negative control group. It has been shown by Cryan et al. [60] that some central excitatory medicines can stimulate the activity and produce an incorrect positive effect. To avoid the possibility of false positive results, the effect of *N. pobeguinii* extracts on the locomotor activity was observed in the OFT. Our findings showed that aqueous and methanol extracts of *N. pobeguinii* did not evoke any important changes in the number of passages and rising, which proposed that the reduced immobility time of mice in both FST and TST induced by both extracts of *N. pobeguinii* had nothing to do with psychostimulant effects [63]. Presently, increase in serotonin and/or noradrenaline neurotransmission is involved in the most current treatment for main depression [64]. Current studies have discovered new visions for the therapeutic role of 5-HT1A receptors in the treatment of numerous CNS illnesses, including depressive disorders [65]. It is also well known that, in the pathophysiology of depression, the dysfunction of the hypothalamic pituitary-adrenal (HPA) axis plays a major role [66]. In fact, a constant activation of the HPA axis can lead to amplified levels of glucocorticoids [67]. Surplus glucocorticoids damage hippocampal neurogenesis function [68], which further causes depression-related illnesses. These findings suggested that the antidepressant effects of N. pobeguinii extracts can be partly facilitated through regulation of neuroendocrine structure or prevention of serotonin reuptake and applied by interrelating through 5-HT1A [60].

Other cognitive dysfunctions associated with neuropathic pain are anxiety disorders. Since it uses visual stimuli, the elevated plus maze is thought to be an etiologically effective animal model of anxiety [69]. It is a well-accepted animal model trial naturally used to test the efficiency of anxiolytic drugs [70]. A precise effect on anxiety is reflected by the percentage of opened/closed areas passed and the time spent [69]. In the present study, oral administration of aqueous and methanol extracts of *N. pobeguinii* (150 and 300 mg/kg) showed an anxiolytic-like effect in STZ-induced diabetic neuropathic mice, since it increased the number of entries and the time spent on open arms and decreased the time spent in closed arms in the EPM test. The social interaction test and light/dark box of anxiety were established to offer an ethologically based test which is sensitive to both anxiolytic and anxiogenic effects [69, 70]. An increase in social interaction is revealing of an anxiolytic consequence, whereas a specific decrease in social interaction specifies an anxiogenic effect. This experiment provides a novel method to estimate the neurobiological mechanisms causing anxiety illnesses [71]. The light/dark box test is founded on the essential dislike of rats for brightly illuminated areas and on the impulsive investigative behavior of rats in response to mild stressors (i.e., a novel environment and light). It has been reported that simple measurement of the time spent in the light area, but not the number of transfers, is the most reliable and useful parameter for assessing an anxiolytic action [72]. Mice treated with aqueous and methanol extracts of *N. pobeguinii* showed increase in the time of social interaction and in the time spent in the light compartment, confirming the effect on the main anxiolytic parameter. The observed anxiolytic effect of *N. pobeguinii* aqueous and methanol extracts may be due to an agonistic effect on GABA/benzodiazepine receptor complex, an antagonistic effect on 5-HT1B receptors, or an agonistic activity on 5-HT1A receptors [73]. *N. pobeguinii* possesses anxiolytic activity similar to diazepam that acts via the GABA receptor complex.

## 5. Conclusion

This study aimed to determine antihyperalgesic, antidepressive, and antianxiety-like effects of aqueous and methanol extracts of *N. pobeguinii* stem-bark in streptozotocin-induced painful neuropathic diabetic mice. The results obtained reveal that these extracts possess antihypernociceptive activities on models of thermal (hot plate) and cold (acetone) allodynia and on models of mechanical (Von Frey) and chemical (formalin) hyperalgesia in a streptozotocin-induced neuropathic pain model. Additionally, these activities have been related to antihyperglycemic, anti-inflammatory, antidepressive, and anxiolytic-like effects. Therefore, for the controlling of neuropathic pain in persons with long-lasting diabetes, aqueous and methanol extracts of *N. pobeguinii* stem-bark may be beneficial as a novel solution.

## Figures and Tables

**Table 1:** Effects of the aqueous and methanol extracts of the *N. pobeguinii* on blood glucose level during 6 weeks after injection of streptozotocin in mice.

Treatment	Dose (mg/kg)	Period (week)						
		0	1	2	3	4	5	6
Normal control	—	106.67 ± 3.24	104.27 ± 2.18	109.07 ± 5.05	107.67 ± 3.62	104.67 ± 3.93	105.57 ± 2.99	106.40 ± 4.01

Diabetic control	—	104.06 ± 2.82	380.20 ± 8.50^*λ*^	440.03 ± 18.27^*λ*^	446.50 ± 17.88^*λ*^	445.03 ± 20.59^*λ*^	448.23 ± 18.13^*λ*^	456.50 ± 14.27^*λ*^

Pregabalin	30	109.67 ± 4.91	381.00 ± 8.65^*λ*^	434.50 ± 24.89^*λ*^	358.33 ± 13.04^*λc*^	354.33 ± 9.28^*λc*^	357.83 ± 13.19^*λc*^	352.13 ± 11.73^*λc*^

Aqueous extract	150	107.67 ± 0.95	376.00 ± 16.17^*λ*^	438.50 ± 16.55^*λ*^	258.31 ± 16.70^*λc*^	168.00 ± 8.75^*c*^	161.67 ± 5.25^*c*^	136.67 ± 6.07^*c*^
	300	104.83 ± 1.49	366.67 ± 10.18^*λ*^	454.34 ± 18.21^*λ*^	224.88 ± 6.88^*λc*^	133.33 ± 5.50^*c*^	124.50 ± 4.28^*c*^	117.50 ± 5.06^*c*^

Methanol extract	150	105.33 ± 1.61	396.00 ± 11.55^*λ*^	448.04 ± 12.59^*λ*^	285.17 ± 4.07^*λc*^	190.02 ± 19.18^*c*^	176.83 ± 11.03^*c*^	140.17 ± 6.47^*c*^
	300	106.83 ± 1.47	391.21 ± 12.57^*λ*^	444.18 ± 16.76^*λ*^	245.19 ± 13.78^*λc*^	161.27 ± 14.06^*c*^	152.18 ± 14.46^*c*^	130.22 ± 8.94^*c*^

All values are expressed as the mean ± SEM of six animals per experimental group, compared by one-way analysis of variance followed by Tukey test; ^*λ*^*p* <0.001 means significantly different from the normal control group (nondiabetic); and ^c^*p* <0.001 means significantly different from the diabetic control group.

**Table 2:** Effects of the aqueous and methanol extracts of the *N. pobeguinii* on body weight during 6 weeks after injection of streptozotocin in mice.

Treatment	Dose (mg/kg)	Period (week)						
		0	1	2	3	4	5	6
Normal control	—	32.00 ± 0.88	34.00 ± 1.57	36.33 ± 0.80	38.00 ± 0.82	38.50 ± 1.50	40.00 ± 0.93	40.77 ± 0.99

Diabetic control	—	35.67 ± 1.52	33.50 ± 0.81	31.17 ± 0.60^*α*^	30.17 ± 0.89^*λ*^	29.67 ± 0.92^*λ*^	28.17 ± 0.79^*λ*^	27.00 ± 0.52^*λ*^

Pregabalin	30	33.50 ± 1.92	31.17 ± 1.28	30.50 ± 1.29^*β*^	29.50 ± 1.15^*λ*^	28.41 ± 1.43^*λ*^	27.83 ± 1.20^*λ*^	26.09 ± 0.73^*λ*^

Aqueous extract	150	34.12 ± 1.69	33.50 ± 0.96	31.17 ± 0.46^*α*^	32.00 ± 0.37^*λ*^	34.22 ± 0.28	34.50 ± 0.22^*βc*^	35.00 ± 0.58^*λc*^
	300	34.51 ± 2.03	33.67 ± 0.81	31.83 ± 0.52	32.17 ± 0.48^*λ*^	34.33 ± 0.25	36.00 ± 0.32^*c*^	36.67 ± 0.42^*βc*^

Methanol extract	150	35.18 ± 1.37	34.17 ± 1.49	30.67 ± 0.60^*β*^	31.67 ± 0.42^*λ*^	33.00 ± 0.68^*α*^	33.67 ± 0.68^*λb*^	34.08 ± 0.58^*λc*^
	300	32.33 ± 2.17	30.19 ± 1.85	29.40 ± 1.74^*λ*^	29.18 ± 1.50^*λ*^	30.67 ± 1.43^*λ*^	31.33 ± 1.33^*λ*^	32.39 ± 1.15^*λc*^

All values are expressed as the mean ± SEM of six animals per experimental group, compared by one-way analysis of variance followed by Tukey test; ^*α*^*p* <0.05, ^*β*^*p* <0.01, ^*λ*^*p* <0.001 mean significantly different from the normal control group (nondiabetic); and ^*b*^*p* <0.01, ^*c*^*p* <0.001 mean significantly different from the diabetic control group.

## Data Availability

All the data supporting our findings are adequately given in the manuscript.

## References

[1] Kiasalari Z., Rahmani T., Mahmoudi N., Baluchnejadmojarad T., Roghani M. (2017). Diosgenin ameliorates development of neuropathic pain in diabetic rats: involvement of oxidative stress and inflammation. *Biomedicine & Pharmacotherapy*.

[2] Neha C., Rajeev T., Pyare L. S. (2012). Effect of dipyrone and thalidomide alone and in combination on STZ-induced diabetic neuropathic pain. *Archives of Pharmacology*.

[3] Ling Q., Liu M., Wu M. X. (2014). Anti-allodynic and neuroprotective effects of koumine, a benth alkaloid, in a rat model of diabetic neuropathy. *The Pharmaceutical Society of Japan*.

[4] Calcutt N. A. (2004). Experimental models of painful diabetic neuropathy. *Journal of the Neurological Sciences*.

[5] Wang Y., Schmeichel A. M., Iida H., Schmelzer J. D., Low P. A. (2006). Enhanced inflammatory response via activation of NF-*κ*B in acute experimental diabetic neuropathy subjected to ischemia-reperfusion injury. *Journal of the Neurological Sciences*.

[6] Silva J. R. L., da Silva M. D. P., Lefort J., Vargaftig B. B. (2000). Endotoxins, asthma, and allergic immune responses. *Toxicology*.

[7] Edwards J. L., Vincent A. M., Cheng H. T., Feldman E. L. (2008). Diabetic neuropathy: mechanisms to management. *Pharmacology & Therapeutics*.

[8] Orrù A., Marchese G., Casu G. (2014). Withania somnifera root extract prolongs analgesia and suppresses hyperalgesia in mice treated with morphine. *Phytomedicine*.

[9] Raison V. M. C. L. (2009). Neurobiology of depression, fibromyalgia and neuropathic pain. *Frontiers in Bioscience*.

[10] McKeage K. (2007). Treatment options for the management of diabetic painful neuropathy: best current evidence. *Current Opinion in Neurology*.

[11] Kellogg A. P., Cheng H. T., Pop-Busui R. (2008). Cyclooxygenase-2 pathway as a potential therapeutic target in diabetic peripheral neuropathy. *Current Drug Targets*.

[12] Hasanein P., Mohammad Zaheri L. (2014). Effects of rosmarinic acid on an experimental model of painful diabetic neuropathy in rats. *Pharmaceutical Biology*.

[13] O’Connor A. B. (2009). Neuropathic pain: quality-of-life impact, costs and cost effectiveness of therapy. *Pharmaco Economics*.

[14] Xu B., Descalzi G., Ye H. R., Zhuo M., Wang Y. W. (2012). Translational investigation and treatment of neuropathic pain. *Journal of Molecular Pain*.

[15] Agnaniet H., Mbot E. J., Keita O. (2016). Antidiabetic potential of two medicinal plants used in Gabonese folk medicine. *BMC Complementary and Alternative Medicine*.

[16] Mesia G. K., Tona G. L., Penge O. (2005). Antimalarial activities and toxicities of three plants used as traditional remedies for malaria in the Democratic Republic of Congo:Croton mubango, Nauclea pobeguiniiandPyrenacantha staudtii. *Annals of Tropical Medicine & Parasitology*.

[17] Mbuta K., Mwima K., Bitengeli M. (2012). *“Plantes médicinales de traditions Province de l’Equateur – R.D.C*.

[18] Luzakibanza Manzo M., Congo D. R. (2012). *Etude phytochimique et pharmacologique de plantes antipaludiques utilisées en médecine traditionnelle Congolaise*.

[19] Betti J. L. (2002). 3Medicinal plants sold in Yaoundé markets, Cameroon. *African Study Monographs*.

[20] Mbiantcha M., Tsafack E. G., Ateufack G. (2018). Analgesic, antiinflammatory and anti-arthritic properties of aqueous and methanolic stem bark extracts from *Nauclea pobeguinii* (Rubiacee) in rats. *Journal of Complementary and Integrative Medicine*.

[21] Tsafack E. G., Djuichou Nguemnang S. F., Atsamo A. D. (2020). In vitro anti-inflammatory, anti-oxidant and in vivo antiarthritic properties of stem bark extracts from Nauclea pobeguinii (Rubiaceae) in rats. *Asian Pacific Journal of Tropical Biomedicine*.

[22] Qi W., Yue S.-J., Sun J.-H., Simpkins J. W., Zhang L., Yuan D. (2014). Alkaloids from the hook-bearing branch ofUncariarhynchophyllaand their neuroprotective effects against glutamate-induced HT22 cell death. *Journal of Asian Natural Products*.

[23] Capasso A., Aquino R., Garofalo L., Simone F., Sorrentino L. (1997). Indole alkaloids from Sickingia williamsii reduce the in-vitro effects of morphine withdrawal in the Guinea-pig. *Journal of Pharmacy and Pharmacology*.

[24] Adjouzem C. F., Ateufack G., Mbiantcha M. (2020). Effects of aqueous and methanolic extracts of stem bark of *Alstonia boonei* de wild. (Apocynaceae) on dextran sodium sulfate-induced ulcerative colitis in wistar rats. *Evidence-Based Complementary and Alternative Medicine*.

[25] Mbiantcha M., Khalid R., Dawe A. (2019). Antihypernociceptive and neuroprotective effects of Combretin A and Combretin B on streptozotocin-induced diabetic neuropathy in mice. *Naunyn-Schmiedeberg’s Archives of Pharmacology*.

[26] Biella G. E. M., Groppetti A., Novelli A., Fernández-Sánchez M. T., Manfredi B., Sotgiu M. L. (2003). Neuronal sensitization and its behavioral correlates in a rat model of neuropathy are prevented by a cyclic analog of orphenadrine. *Journal of Neurotrauma*.

[27] Mbiantcha M., Khalid R., Atsamo D. A. (2020). Anti-hypernociceptive effects of methanol extract of *Boswellia dalzielii* on STZ-induced diabetic neuropathic pain. *Advances in Traditional Medicine*.

[28] Gutierrez T., Nackley A. G., Neely M. H., Freeman K. G., Edwards G. L., Hohmann A. G. (2003). Effects of neurotoxic destruction of descending noradrenergic pathways on cannabinoid antinociception in models of acute and tonic nociception. *Brain Research*.

[29] Fatani A. J., Al-Rejaie S. S., Abuohashish H. M. (2015). Neuroprotective effects of *Gymnema sylvestre* on streptozotocin-induced diabetic neuropathy in rats. *Experimental and Therapeutic Medicine*.

[30] Felipe F. C. B., Filho J. T. S., Souza L. O. (2007). Piplartine, an amide alkaloid from Piper tuberculatum, presents anxiolytic and antidepressant effects in mice. *Phytomedicine*.

[31] Bhattamisra S. K., Singh P. N., Singh S. K., Kumar V. (2007). Anxiolytic activity of marsilea minuta linn. *Journal of Herbal Medicine and Toxicology*.

[32] File S. E. (1996). The use of social interaction as a method for detecting anxiolytic activity of chlordiazepoxide like drugs. *Journal of Neuroscience Methods*.

[33] Edwards J. L., Vincent A., Cheng T., Feldman E. L. (2008). Diabetic neuropathy: mechanisms to management. *Pharmacology & Therapeutics*.

[34] Jolivalt C. G., Lee C. A., Ramos K. M., Calcutt N. A. (2008). Allodynia and hyperalgesia in diabetic rats are mediated by GABA and depletion of spinal potassium-chloride co-transporters. *Pain*.

[35] Jason D. F., Camilla I. S., Annika B. M., Calcutt A. (2002). Elevated spinal cyclooxygenase and prostaglandin release during hyperalgesia in diabetic rats. *Diabetes*.

[36] Zychowska M., Rojewska E., Kreiner G., Nalepa I., Przewlocka B., Mika J. (2013). Minocycline influences the anti-inflammatory interleukins and enhances the effectiveness of morphine under mice diabetic neuropathy. *Journal of Neuroimmunology*.

[37] Niels M., Sreekumaran N. (2008). Diabetes and protein metabolism. *Diabetes*.

[38] Balamurugan K., Nishanthini A., Molan R. (2014). Antidiabetic and antihyperlipidaemic activity of ethanol extract of *Melastoma malabathricum* Linn. Leaf in alloxan induced diabetic rats. *Asian Pacific Journal of Tropical Biomedicine*.

[39] Wilson N. M., Wright D. E. (2011). Inflammatory mediators in diabetic neuropathy. *Journal of Diabetes and Metabolism*.

[40] Mika J., Osikowicz M., Rojewska E. (2009). Differential activation of spinal microglial and astroglial cells in a mouse model of peripheral neuropathic pain. *European Journal of Pharmacology*.

[41] Daulhac L., Mallet C., Courteix C. (2006). Diabetes-induced mechanical hyperalgesia involves spinal mitogen-activated protein kinase activation in neurons and microglia via N-Methyl-D-aspartate-Dependent mechanisms. *Molecular Pharmacology*.

[42] Tsuda M., Ueno H., Kataoka A., Tozaki-Saitoh H., Inoue K. (2008). Activation of dorsal horn microglia contributes to diabetes-induced tactile allodynia via extracellular signal-regulated protein kinase signaling. *Glia*.

[43] Watkins L. R., Hutchinson M. R., Ledeboer A., Wieseler-Frank J., Milligan E. D., Maier S. F. (2007). Glia as the “bad guys”: implications for improving clinical pain control and the clinical utility of opioids. *Brain, Behavior, and Immunity*.

[44] Zuo Y., Perkins N. M., Tracey D. J., Geczy C. L. (2003). Inflammation and hyperalgesia induced by nerve injury in the rat: a key role of mast cells. *Pain*.

[45] Sweitzer S. M., Hickey W. F., Rutkowski M. D., Pahl J. L., Deleo J. A. (2002). Focal peripheral nerve injury induces leukocyte trafficking into the central nervous system: potential relationship to neuropathic pain. *Pain*.

[46] Watson J. J., Allen S. J., Dawbarn D. (2008). Targeting nerve growth factor in pain. *BioDrugs*.

[47] Mantyh P. W., Koltzenburg M., Mendell L. M., Tive L., Shelton D. L., Warner D. S. (2011). Antagonism of nerve growth factor-trkA signaling and the relief of pain. *Anesthesiology*.

[48] Dyck P. J., Peroutka S., Rask C. (1997). Intradermal recombinant human nerve growth factor induces pressure allodynia and lowered heat-pain threshold in humans. *Neurology*.

[49] Kuete V., Sandjo L. P., Mbaveng A. T., Seukep J. A., Ngadjui B. T., Efferth T. (2015). Cytotoxicity of selected Cameroonian medicinal plants and Nauclea pobeguinii towards multi-factorial drug-resistant cancer cells. *BMC Complementary and Alternative Medicine*.

[50] Zhu H., Liang Q., Xiong X. (2018). Anti-inflammatory effects of p-coumaric acid, a natural compound of *Oldenlandia diffusa*, on arthritis model rats. *Evidence-Based Complementary and Alternative Medicine*.

[51] Tao L., Ding Q., Gao C., Sun X. (2016). Resveratrol attenuates neuropathic pain through balancing pro-inflammatory and anti-inflammatory cytokines release in mice. *International Immunopharmacology*.

[52] Yar A. S., Menevse S., Alp E., Helvacioglu F., Take G. (2009). The effects of resveratrol on cyclooxygenase-1 and cyclooxygenase-2 mRNA and protein levels in diabetic rat kidneys. *Molecular Biology Reports*.

[53] Subbaramaiah K., Chung W. J., Michaluart P. (1998). Resveratrol inhibits cyclooxygenase-2 transcription and activity in phorbol ester-treated human mammary epithelial cells. *Journal of Biological Chemistry*.

[54] Zhao Y., Liu J. (2016). Anti-inflammatory effects of *p*-coumaric acid in LPS-stimulated RAW264.7 cells: involvement of NF-*κ*B and MAPKs pathways. *Medicinal Chemistry*.

[55] Santos J. A., Piccinelli A. C., Formagio M. D. (2017). Antidepressive and antinociceptive effects of ethanolic extract and fruticuline A from salvia lachnostachys Benth leaves on rodents. *PLoS One*.

[56] Radat F., Koleck M. (2011). Douleur et dépression: les médiateurs cognitifs et comportementaux d’une association très fréquente. *L’encephale*.

[57] Xu Y. J., Foubert K., Dhooghe L., Lemiere F., Cimanga K., Mesia K. (2012). Chromatographic profiling and identification of two new iridoid-indole alkaloids by UPLC-MS and HPLC-SPE-NMR analysis of an antimalarial extract from Nauclea pobeguinii. *Planta Med*.

[58] Wang J., Goffer Y., Xu D. (2011). A single subanesthetic dose of ketamine relieves depression-like behaviors induced by neuropathic pain in rats. *Anesthesiology*.

[59] Chang C. Y., Guo H. R., Tsai W. C. (2015). Subchronic arsenic exposure induces anxiety-like behaviors in normal mice and enhances depression-like behaviors in the chemically induced mouse model of depression. *BioMed Research International*.

[60] Cryan J. F., Mombereau C., Vassout A. (2005). The tail suspension test as a model for assessing antidepressant activity: review of pharmacological and genetic studies in mice. *Neuroscience & Biobehavioral Reviews*.

[61] Dulawa S. C., Holick K. A., Gundersen B., Hen R. (2004). Effects of chronic fluoxetine in animal models of anxiety and depression. *Neuropsychopharmacology*.

[62] D Shalam M., Shantakumar S. M., Narasu M. L. (2007). Pharmacological and biochemical evidence for the antidepressant activity of the herbal preparation trans-01. *Indian Journal of Pharmacology*.

[63] Li Y.-C., Shen J.-D., Li Y.-Y., Huang Q. (2014). Antidepressant effects of the water extract fromTaraxacum officinaleleaves and roots in mice. *Pharmaceutical Biology*.

[64] Brunello N., Blier P., Judd L. L. (2003). Noradrenaline in mood and anxiety disorders: basic and clinical studies. *International Clinical Psychopharmacology*.

[65] Prieto E., Micó2 J. A., Meana J. J., Majadas S. (2010). Neurobiological bases of quetiapine antidepresant effect in the bipolar disorder. *Actas Españolas de Psiquiatría*.

[66] Risbrough V. B., Stein M. B. (2006). Role of corticotropin releasing factor in anxiety disorders: a translational research perspective. *Hormones and Behavior*.

[67] Stranahan A. M., Arumugam T. V., Cutler R. G., Lee K., Egan J. M., Mattson M. P. (2008). Diabetes impairs hippocampal function through glucocorticoid-mediated effects on new and mature neurons. *Nature Neuroscience*.

[68] Zhang W.-J., Tan Y.-F., Yue J. T. Y., Vranic M., Wojtowicz J. M. (2008). Impairment of hippocampal neurogenesis in streptozotocin-treated diabetic rats. *Acta Neurologica Scandinavica*.

[69] Patro G., Bhattamisra S. K., K Mohanty B. (2016). Effects of *Mimosa pudica* L. leaves extract on anxiety, depression and memory. *Avicenna Journal of Phytomedicine*.

[70] Larissa Fernanda D. A. V., Maria Danielma D. S. R., Altair Rogério A. B. (2013). Anxiolytic-like effect of the extract from Bowdichia virgilioides in mice. *Brazilian Journal of Pharmacognosy*.

[71] Kumar D., Bhat Z. A., Kumar V., Khan N. A., Chashoo I. A., Zargar M. I. (2012). Effects of Stachys tibetica essential oil in anxiety. *European Journal of Integrative Medicine*.

[72] Wei X.-Y., Yang J.-Y., Wang J.-H., Wu C.-F. (2007). Anxiolytic effect of saponins from Panax quinquefolium in mice. *Journal of Ethnopharmacology*.

[73] Thippeswamy B., Mishra B., Veerapur V., Gupta G. (2011). Anxiolytic activity of Nymphaea alba Linn. in mice as experimental models of anxiety. *Indian Journal of Pharmacology*.

